# Metabolic Effects of Sucralose on Environmental Bacteria

**DOI:** 10.1155/2013/372986

**Published:** 2013-12-03

**Authors:** Arthur Omran, Gregory Ahearn, Doria Bowers, Janice Swenson, Charles Coughlin

**Affiliations:** Department of Biology, University of North Florida, Jacksonville, FL 32224, USA

## Abstract

Sucralose was developed as a low cost artificial sweetener that is nonmetabolizable in humans. Sucralose can withstand changes in pH and temperature and is not degraded by the wastewater treatment process. Since the molecule can withstand heat, acidification, and microbial degradation, it is accumulating in the environment and has been found in wastewater, estuaries, rivers, and the Gulf Stream. Environmental isolates were cultured in the presence of sucralose looking for potential sucralose metabolism or growth acceleration responses. Sucralose was found to be nonnutritive and demonstrated bacteriostatic effects on all six isolates. This growth inhibition was directly proportional to the concentration of sucralose exposure, and the amount of the growth inhibition appeared to be species-specific. The bacteriostatic effect may be due to a decrease in sucrose uptake by bacteria exposed to sucralose. We have determined that sucralose inhibits invertase and sucrose permease. These enzymes cannot catalyze hydrolysis or be effective in transmembrane transport of the sugar substitute. Current environmental concentrations should not have much of an effect on environmental bacteria since the bacteriostatic effect seems to be consecration based; however, as sucralose accumulates in the environment, we must consider it a contaminant, especially for microenvironments.

## 1. Introduction

Sucralose was the first noncalorie sweetener made from natural sugar, being manufactured by the selective chlorination of sucrose, which substitutes three of the hydroxyl groups with chlorines [1]. Sucralose is stable under increased heat and over a broad range of acidic and alkaline conditions. Therefore, sucralose can be used in baking or in products that require a longer shelf-life [2]. Sucralose causes exactly zero caloric increase in mammals [1]. Artificial sweeteners have been considered contaminants by environmental scientists only recently [3]. Due to the human inability to metabolize these molecules, they are passed on to the environment via human excrement, and the highest concentration (2,800 ± 1,000 ng/L) of combined artificial sweetener contaminants is found in wastewater treatment reservoirs [4]. Artificial sweeteners such as saccharin and cyclamates are found mostly degraded by the wastewater treatment process. Sucralose, however, is found in higher concentrations and was degraded minimally [4]. Degradation only occurs to a limited extent during hydrolysis, ozonation, and microbial processes indicating that breakdown of sucralose will likely be slow and incomplete leading to accumulation of sucralose in surface waters [5]. Sucralose has been detected in rivers in North Carolina, in the Gulf Stream, and in the waters of the Florida Keys [6]. Scientists are detecting sucralose in various U.S. inland surface waters and monitoring its accumulation [4]. Most artificial sweeteners are either partially or completely broken down due to the wastewater treatment process using high temperatures and changes in pH and constant filtration. It would seem that the ability of sucralose to withstand drastic pH and temperature changes makes it an exception among artificial sweeteners [5]. Over time sucralose may spread to other aquatic and coastal ecosystems, increasing in concentration [5]. These researchers also speculated that the persistent qualities of sucralose may lead to chronic low-dose exposure with largely unknown consequences for human and environmental health.

Sucralose's effect on environmental microbes is largely unknown. However, studies of human oral and gut bacteria have shown an inhibition of bacterial growth in the presence of sucralose [7]. In one study the incorporation of 126 mM sucralose into glucose agar medium caused total inhibition of growth of *Streptococcus sobrinus* 6715-17, *Streptococcus sanguis* 10904, *Streptococcus challis*, *Streptococcus salivarius*, and *Actinomyces viscosus* WVU627 [7]. In a related study rats were infected with *Streptococcus sobrinus* and, following a sucrose water diet, developed dental caries lesions [8]. Another group of rats given the same bacteria but sucralose water instead of sugar water had a significant decrease in caries lesions in their teeth. These researchers concluded that oral bacteria cannot grow on the artificial sweetener hence causing less damage, indicating sucralose is noncariogenic [8]. The same inhibition may be true for environmental microbes. Furthermore, if sucralose does inhibit bacterial growth, the type of inhibition would need to be identified as either bactericidal (killing the bacteria) or bacteriostatic (slowing bacterial metabolism), and the mechanisms of such inhibition should be elucidated.

## 2. Methods and Materials

### 2.1. Summary of Methodology

To elucidate the effect of sucralose on bacterial growth, bacterial isolates were sampled from diverse environments. Each bacterium isolated was surveyed for sucralose metabolism. If sucralose was found to be nonnutritive for the bacterium, the effect on healthy bacterial growth was observed via turbidity testing by culturing the bacterial isolates on TSB and amending the media with various concentrations of sucralose. Any inhibition was typed as either bacteriostatic or bactericidal; this was determined with a disk diffusion assay and reculturing. Finally the mechanism of such inhibitory effect was identified by enzyme and transport assays. Based on the molecular kinetics analysis the type of inhibition was determined. Transport inhibition and reduction in catalysis could be indicators of competitive inhibition.

### 2.2. Growth/Turbidity Testing

Six individual isolates were cultured in Tryptic Soy Broth (*n* = 5) (TSB) (Difco Laboratories, MI, USA) and incubated at 25°C. The control group consisted of 5 mL of TSB amended with additional 0.5 mL growth medium. The experimental groups included 5 mL TSB with 0.5 mL of 10, 20, 30, or 40% by volume sucralose added, yielding final concentrations of 27.8 mM, 55.78 mM, 83.75 mM, and 111.7 mM. Turbidity of the cultures was measured at 620 nm once daily for 9 days using 24-hour intervals (Sequoia Turner 340 Spectrophotometer).

### 2.3. Sucralose Metabolism Validation

Individual isolates were cultured in M9 broth medium (*n* = 6) (TechNova, NS, Canada) and incubated at 25°C. The control group consisted of 5 mL of M9 broth without a carbon source; the experimental group included a 5 mL of M9 broth with sucralose as the only carbon source. Turbidity of the cultures was measured over the next 9 days using 24-hour time intervals and a Sequoia Turner Spectrophotometer set to 620 nm wavelength.

### 2.4. Disk Diffusion Assay and Determination of the Type of Inhibition

Each bacterial isolate was spread-plated onto a TSA medium. Disks were prepared by hole punching out cotton rounds, which were soaked with 1.6 M sucralose. The disks were then placed onto the surface of the media, 3 disks per Petri dish (*n* = 9). Samples were incubated over night at 25°C, and diameters of the zones of inhibition were measured. The zones of inhibition were then swabbed and used to inoculate new TSA media. These reculture plates were incubated over night at 25°C and then inspected for growth.

### 2.5. Cell Death Assay

Each isolate was individually cultured onto six M9 agar plates with glucose and six M9 agar plates with sucrose. Threefold serial dilutions of stock cultures were made and spread-plated; then 350 *μ*L of 25.1 mM sucralose was poured onto the surface of each of the sucralose added groups shortly after inoculation. These were incubated at 25°C for 48 hours, and then the plates were inspected and colonies counted. The isolate that exhibited the greatest percentage of cell death on the M9 sucrose medium compared to M9 glucose medium was selected for transport inhibition testing in order to elucidate an inhibitory mechanism.

### 2.6. Transport Inhibition Assay

*Streptomyces badius* was selected for transport inhibition analysis. This was due to the significant degree of growth inhibition on M9 sucrose medium challenged with sucralose. A Bradford Coomassie assay was conducted to standardize protein concentration. From this, appropriate cell culture concentrations were selected in order to yield the appropriate amount of total protein for transport. Three test groups were used to measure potential transport inhibition: (1) a 0.1 mM sucrose only group; (2) a 0.1 mM sucrose and 0.1 mM sucralose group; and (3) a 0.1 mM sucrose and 0.1 mM mannitol group which served as a control to ensure that osmotic shock was not occurring during the transport test. Each group contained 700 *μ*L of diluted M9 salt aliquots (64 g Na_2_HPO_4_, 15 g KH_2_PO_4_, 2.5 g NaCl, and 5 g NH_4_Cl per 5 liters H_2_O) and 0.5 *μ*L of ^14^C-sucrose (0.41 *μ*Ci/pmole). Lastly 300 *μ*L of cell culture in stationary growth phase was extracted and placed into the mixture and shaken vigorously. The contents of the reaction tubes were incubated at 25°C for 2 minutes. Contents of the culture tubes were then filtered onto 0.45 *μ*m pore size filter disks and washed with 2 mL of stop solution (ice cold M9 salt aliquots). Filter disks were placed into a tube with scintillation fluid and the radioactivity was measured via a Beckman 6500 scintillation counter.

### 2.7. Enzyme Kinetics: Invertase Inhibition Assay

Stock solutions were prepared of 0.3 U/L invertase, 1.5 M sucrose, 1 M sucralose, and standard Benedict's solution. Two test groups were prepared: (1) a sucrose only set and (2) a sucrose and sucralose set. The sucrose only set had 6 reaction tubes prepared. Each reaction tube contained 1 mL 0.3 U/L of invertase, 0.25 mL of Benedict's solution, and 0.75 mL pH 4 buffer. Each tube in the set contained different amounts of sucrose: 2.5 mM, 5 mM 10 mM, 15 mM 20 mM, and 25 mM. Reaction tubes were incubated at 75°C for 30 minutes; the absorbance of each tube was measured at 485 nm on a Sequoia Turner 340 Spectrophotometer every 2 minutes. At minute 5 the initial velocity was recorded. The sucrose and sucralose set had 6 reaction tubes prepared. Each reaction tube contained 1 mL 0.3 U/L of invertase, 0.25 mL of Benedict's solution, 0.75 mL pH 4 buffer, and 0.55 mM sucralose. Each tube contained a different concentration of sucrose, 2.5 mM, 5 mM 10 mM, 15 mM 20 mM, and 25 mM sucrose concentrations, respectively. Reaction tubes were incubated at 75°C for 30 minutes; the absorbance of each tube was measured at 485 nm on a Sequoia Turner 340 Spectrophotometer every 2 minutes. At minute 5 the initial velocity was recorded. Once these assays were completed, the velocities were analyzed and used to generate an enzyme kinetics plot to determine the type of inhibition sucralose exerted on invertase.

## 3. Results/Discussion

**Figure 1:**
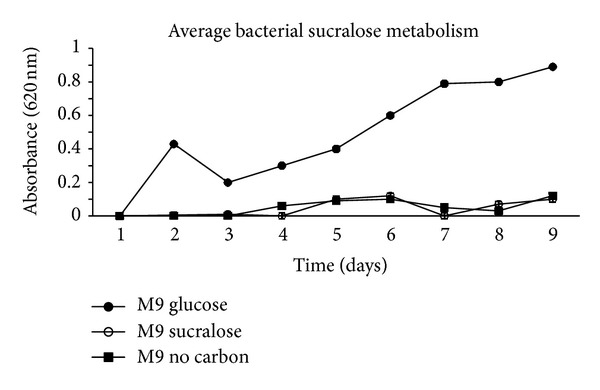
A composite growth curve depicting average bacterial growth with various carbon sources of the 6 isolates. M9 medium containing glucose as the only carbon source serves as a positive control, M9 medium containing only sucralose as a carbon source was the experimental group, and M9 medium containing no carbon source serves as a “starvation diet” or negative control. This was done to indicate the presence, if any, of sucralose metabolism.

All 6 isolates had fewer colony forming units (CFUs) on the media exposed to sucralose than they had on the positive control groups of M9 sucrose and M9 glucose (Figure 3) indicating an inhibitory effect. Organisms did not metabolize sucralose as shown in Figure 1, indicating that sucralose is nonnutritive for bacteria.

**Figure 2:**
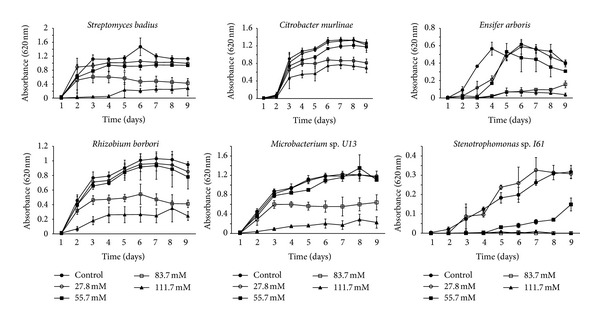
Growth curves for environmental isolates. Each isolate was cultured in TSB amended with varying concentrations of sucralose. The open circle indicates the positive control, closed circle is the 27.8 mM group, the closed square is the 55.3 mM group, the open square is the 83.7 mM group, and the closed triangle is the 111.7 mM group. The positive control group consisted of no sucralose added to the TSB in order to ascertain normal growth. This was performed to determine the effect that sucralose had on bacterial growth. The 83.7 mM and 111.7 mM concentrations were significantly (*P* < 0.05) inhibited compared to the control group for each isolate. The 55.3 mM concentration was significantly (*P* < 0.05) inhibited compared to the control group for *Stenotrophomonas* sp. and *Ensifer arboris*.

To elucidate the effect of sucralose on bacterial growth, turbidity testing was performed. The isolates were subcultured (*n* = 5) in the presence of 27.8 mM, 55.78 mM, 83.75 mM, and 111.7 mM sucralose to further elucidate the effects of sucralose on bacterial growth. Growth curves revealed a decrease in growth with those cultures receiving sucralose compared to the controls which received no sucralose (Figure 2). In general, the greater the sucralose concentration the bacteria were exposed to, the lower the rate of bacterial growth. Not all concentrations were inhibitory. The least concentrated dilution (28.7 mM) showed no inhibitory effects on any of the six bacterial isolates. The 55.7 mM sucralose had minor inhibition on the isolates and was significantly different (*P* < 0.001) for only 2 of the isolates. Standard error of the means indicates a significant difference (*P* < 0.001) between control groups and experimental groups amended with 83.75 mM and 111.7 mM sucralose. One way ANOVA testing on final data points for each treatment revealed significant differences (*P* < 0.001) between the control groups and experimental groups amended with 83.75 mM and 111.7 mM sucralose. These results indicate the effect of sucralose as a growth inhibitor for bacteria from diverse genera.

Disk diffusion assays (*n* = 9) exhibited a wide range of zones of inhibition with species responses being different. Inhibition was considered significant. Each clear zone was sampled and used to inoculate a fresh culture dish. Regrowth indicated a bacteriostatic effect, with all clear zones sampled yielding growth. Regrowth was of the same colony morphology and Gram character as the original culture for each isolate. This result suggests that sucralose is not a bactericidal agent (Table 1).

**Figure 3:**
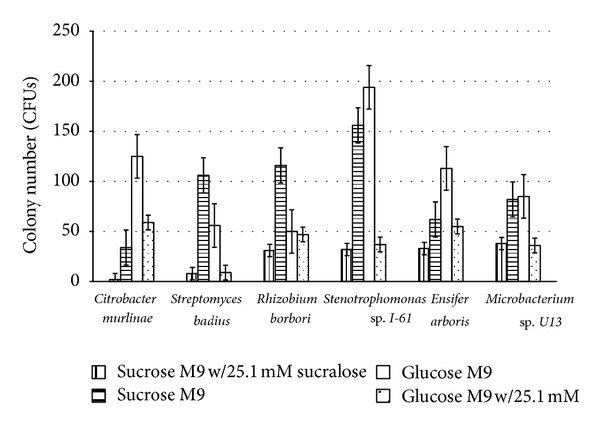
Cell death graph for comparison of inhibition on different carbon source media. Each isolate was cultured in equimolar (111 mM) amounts of either sucrose or glucose as their carbon source, with half the samples also containing sucralose. Finally colony counts were performed.

To elucidate the mechanism of the bacteriostatic inhibition, differential growth effects on normal carbon sources while exposed to sucralose were utilized. Bacterial isolates were partially inhibited when cultured on glucose M9 agar with sucralose and on sucrose M9 agar with sucralose. The colony counts for the media containing sucralose were consistently lower than those of media free of sucralose (Figure 3). *Streptomyces badius* showed significant inhibition (*P* < 0.001) on sucralose containing media. This inhibition was greater than in other isolates, and greater inhibition was observed on sucrose M9 medium than on glucose M9 medium. Standard error of the means was utilized to test to see if this inhibition was significant. Therefore, *Streptomyces badius* was utilized for transport testing (Figure 3).

**Figure 4:**
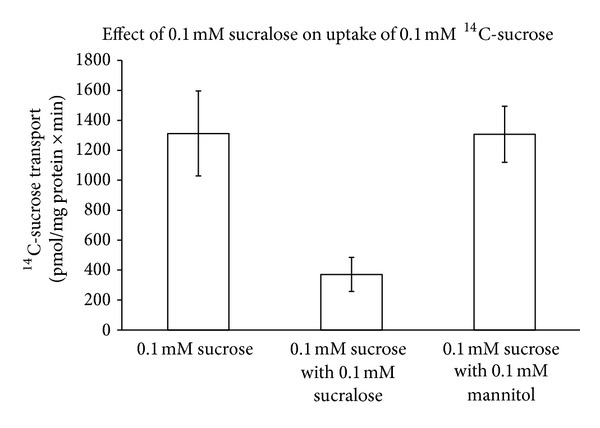
Transport inhibition data: pmol/(mg protein × min) for *Streptomyces badius*. This suggests that sucralose is an inhibitor of sucrose uptake via transport proteins in *S. badius*. The columns are means ± 1SEM (*n* = 5).

A novel transport test was developed to test this phenomenon. A 0.1 mM mannitol control was used to ensure that the effects of sucralose were not due to osmotic shock. Mannitol was used because it has the same osmolar effect that sucralose has in liquid media. A two-tailed *t*-test indicated that there was a significant decrease (*P* < 0.01) in transport of C^14^ labeled sucrose by *Streptomyces badius* when exposed to sucralose (Figure 4).

**Figure 5:**
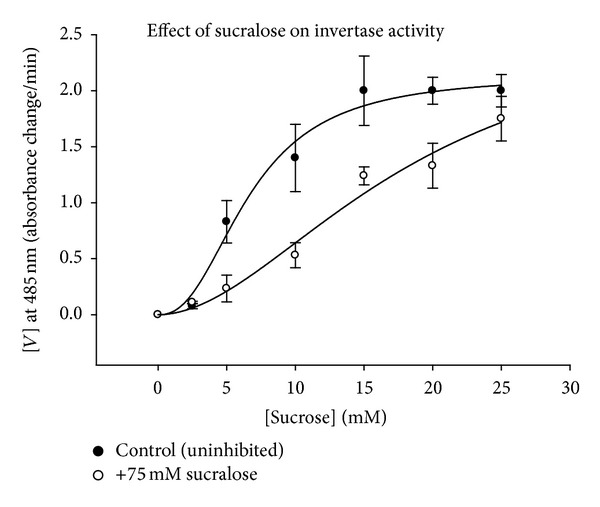
An enzyme kinetics graph of the initial velocities of uninhibited invertase reaction and invertase inhibited with sucralose. The overlapping *V*_max⁡_ values but different *K*_*m*_ values for the reactions indicate competitive inhibition. The error bars are means ± 1SEM (*n* = 5).

To further glean a molecular mechanism of inhibition, enzyme assays were conducted using invertase to catalyze sucrose degradation [9]. Invertase was selected due to its conserved and broad usage in the microbial world, being found in bacteria and fungi. It has been found throughout the domain Bacteria: in extremophiles, gut flora, and environmental bacterial species and has a high percent of homology between clades (9; 10; 11; 12). Invertase is an enzyme that catalyzes the breakdown of sucrose into glucose and fructose. The initial reaction rate and overall reaction rate of invertase were inhibited when the enzyme was suspended in solutions containing sucralose (Figure 5). This shows that sucralose was an inhibitor of invertase enzymatic activity. The kinetics plot was prepared using initial velocities of the uninhibited reaction with inhibited reactions at equimolar concentrations of sucrose [9] (Figure 5). The results of two-tailed *t*-tests for the kinetics study revealed *V*_max⁡_ values that were not significantly different (*P* > 0.05) but *K*_*m*_ values for the reactions that were significantly different (*P* < 0.05) (Figure 5; Table 2). This is indicative of competitive inhibition between sucrose and sucralose for binding to invertase, with sucrose having the higher binding affinity, which is why the inhibitory affect is concentration-based.

Sucralose reducing sucrose uptake and breakdown in bacteria by competing for a binding site serves as a potential mechanism for the bacteriostatic effect observed during growth trials. In previous dental studies sucralose caused oral bacteria to proliferate less frequently, preventing cavity formation [10]. Other studies also noted that the lab mice given sucralose had less detectable fecal bacteria and that gut bacteria were inhibited by sucralose [11]. Also previous environmental microbiological studies indicated inhibition of bacterial growth by sucralose [12]. It is possible that the inhibition in previous studies was bacteriostatic, and these oral and gut bacterial tests are in concurrence with the environmental bacterial testing results presented in this communication.

The main conclusion of this study is that sucralose is an environmental contaminant. It will accumulate in aquatic environments over time because it is not likely to break down (that would require bacterial metabolism). Previous studies suggest that bacterial consortia can partially metabolize sucralose into a di-chloro-aldehyde form; however, these studies indicate that the carbon from sucralose is not incorporated into the bacterial consortium's biomass [13]. This means that the consortium did not fully digest the sucralose, and these studies also point out that the members of their bacterial consortium could not individually metabolize sucralose as a carbon source [14].

The current environmental levels of sucralose (around an average of 300 ng/L depending on location) may not have any effect on bacterial growth. We postulate that current environmental concentrations of sucralose are too low to be having an effect on environmental bacteria in high volume aquatic environments. Bacteria living in microenvironments may experience growth inhibition due to the varying concentrations of water within the microenvironment.

Sucralose is, however, increasing in its concentration due to its inability to be degraded by pH and temperature changes [4]. It is presently in wastewater effluents at levels of several *μ*g/L (ppb). The Swedish Environmental Protection Agency warns that its breakdown is slow and the ecological impact is largely unknown; they emphasize that certain concentration levels may lead to damaging arthropod and cyanobacteria communities [15].

Sucralose inhibition is bacteriostatic and concentration-based. The present sucralose environmental concentrations are too low to negatively affect bacteria presently living in freshwater or soil systems. The concentration of sucralose in these environments is increasing over time [4]. Microenvironments could experience inhibition due to sucralose buildup as these environments may have limited water volumes.

## Figures and Tables

**Table 1:** Disk diffusion assay data; zones of inhibition are indicated. Regrowth from inhibited zones was tested (*n* = 9); regrowth indicated a bacteriostatic inhibition not bactericidal.

Isolate	Inhibition +/−	Regrowth
*M*. sp. U 13	+	yes
*S*. sp. I_61	+	yes
*R. borbori *	+	yes
*C. murlinae *	+	yes
*E. arboris *	+	yes
*S. badius *	+	yes

**Table 2:** Invertase reaction rate kinetic constants from Figure 5. V_max⁡_ units in change in absorbance per minute and Km in mM. The values are means +/− 1SEM (*n* = 5).

	K_*m*_	V_max⁡_	Hill coefficient
Positive control	6.66 ± 0.842	2.13 ± 0.16	2.37 ± 0.56
75 mM sucralose added	18.52 ± 9.78	2.69 ± 1.25	1.88 ± 0.65
